# Supplementary material for: When cheating turns into a stabilizing mechanism of plant–pollinator communities
Source: PLoS Biol. 2023 Dec 27;21(12):e3002434. doi: 10.1371/journal.pbio.3002434 (PMC10752559; doi:10.1371/journal.pbio.3002434)
Supplement: S1 Results — (PDF) [file pbio.3002434.s002.pdf]

Supplementary Materials for

When cheating turns into a stabilizing mechanism of mutualistic networks
François Duchenne, Stéphane Aubert, Elisa Barreto, Emanuel Brenes, Maria Alejandra Maglianesi, Tatiana Santander, Esteban A. Guevara & Catherine H. Graham
Correspondence to: francois.duchenne@wsl.ch

This PDF file includes

Supplementary figures 2
Supplementary tables 11

Supplementary figures

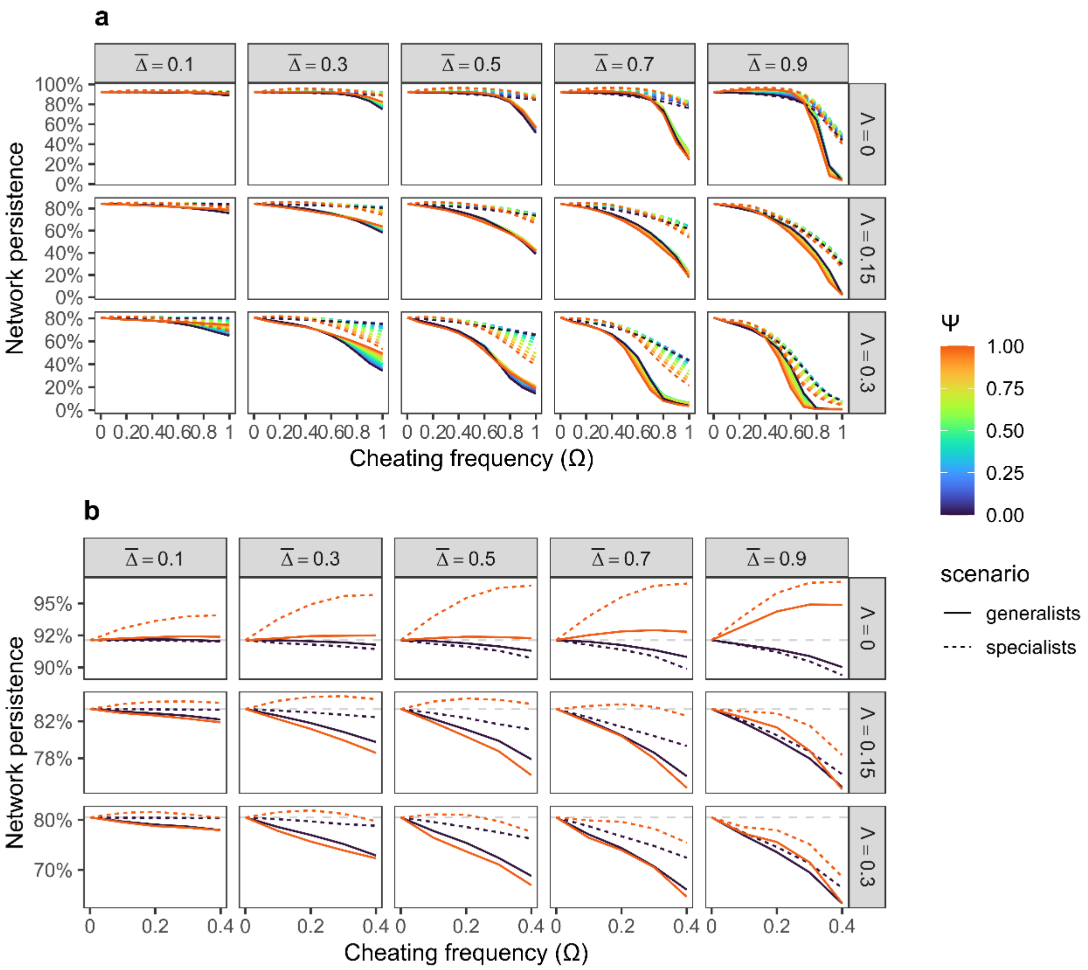

Fig A: Innovative cheating can increase network persistence. *(a) Network persistence as a function of cheating frequency (Ω), proportion of cheaters ($\bar{\Delta}$), cost associated with mutualism (Λ) and proportion of innovative cheating (Ψ). Network persistence is measured as percentage of species (plants and pollinators) having positive abundance at equilibrium, relative to the total initial number of species. Dashed lines show average results when cheaters are the most specialized species and solid lines when cheaters are the most generalist species. Here results are presented for initial values of connectance φ=0.4 and averaged over the 500 initial conditions. (b) Shows the same than in (a) but on a truncated axis of cheating frequency and for two extremes values only, when cheating is conservative only (Ψ=0) or innovative only (Ψ=1). The dashed grey line shows the average network persistence when there was no cheating. Here results are presented for initial values of connectance φ=0.4 and averaged over the 500 initial conditions. A cost of Λ=0.15 and Λ=0.3, corresponds to 10% and 20% of benefits associated with mutualism, respectively (Λ/α = 0.1 and 0.2). The data underlying this Figure can be found in* https://doi.org/10.5281/zenodo.10102438.

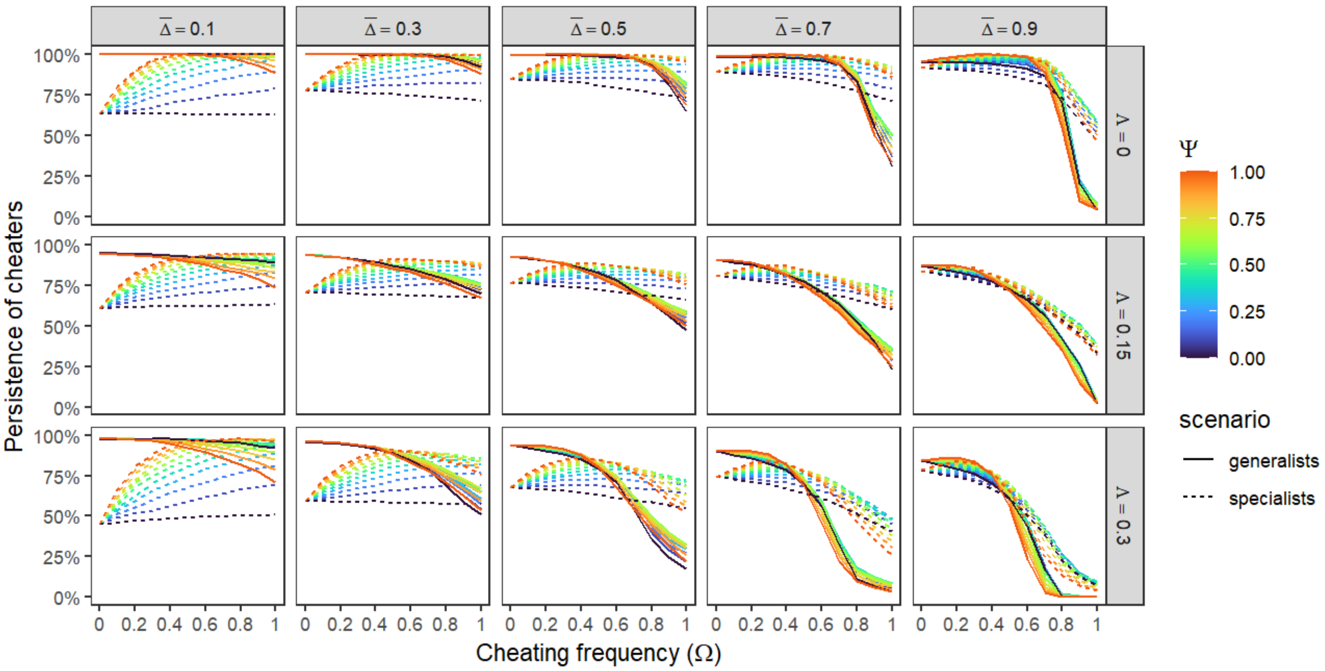

Fig B: Effects of cheating on cheater persistence depend on the generalism degree of the cheaters. *Cheater persistence (i.e. number of initial cheaters divided by number of cheater at equilibrium) as a function of cheating frequency (Ω), proportion of cheaters ($\bar{\Delta}$), cost associated with mutualism (Λ) and proportion of innovative cheating (Ψ). Dashed lines show average results when cheaters are the most specialized species and solid lines when cheaters are the most generalist species. Here results are presented for initial values of connectance φ=0.4 and averaged over the 500 initial conditions. We can see that for low and intermediate level of cheating, innovative cheating tends to increase the persistence of cheaters, if they are specialists, while it is the opposite if they are generalist. The data underlying this Figure can be found in https://doi.org/10.5281/zenodo.10102438.*

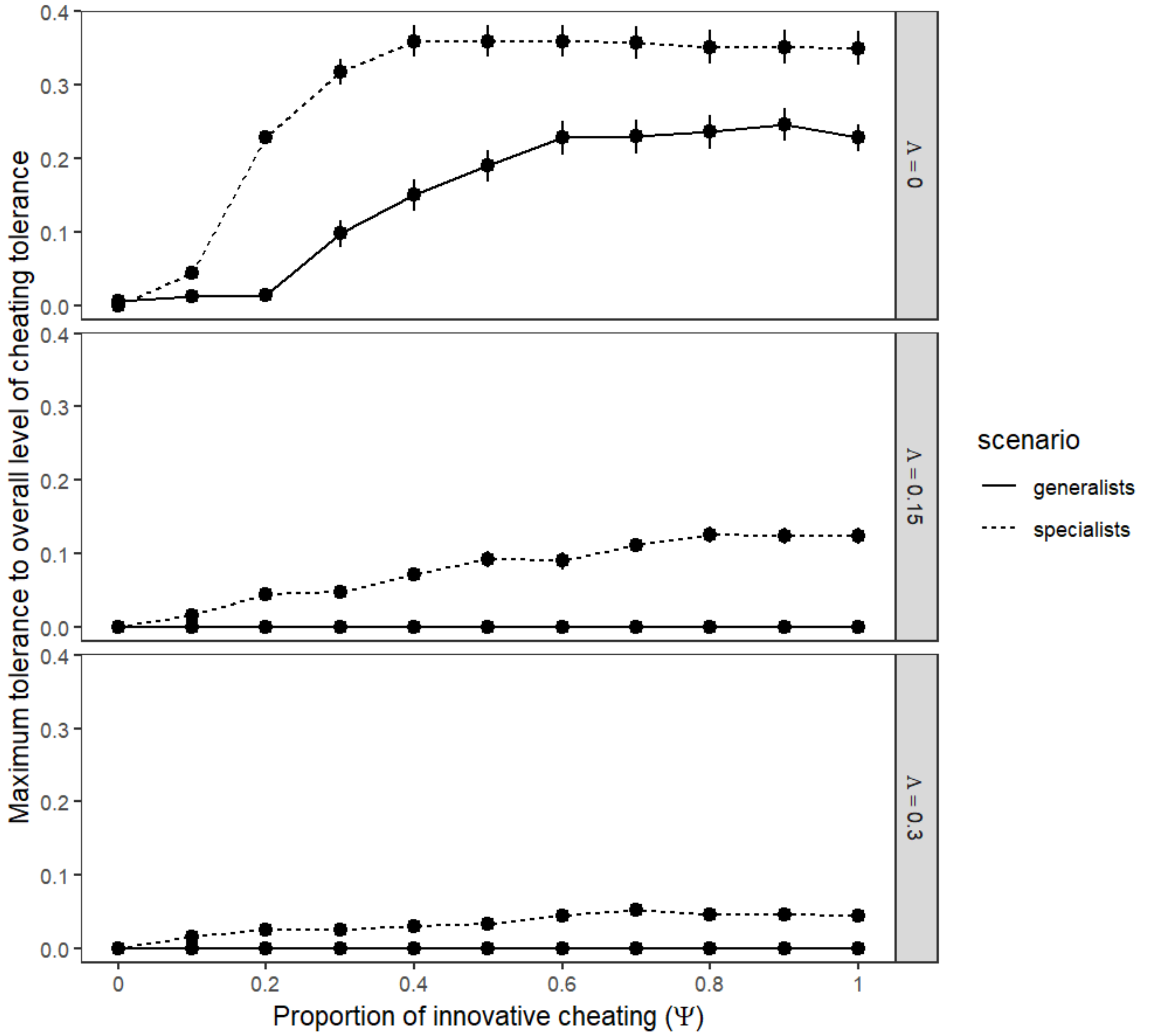

Figure C: Maximum overall level of cheating allowing positive effects on persistence increases when cheating is innovative but decreases with cost associated with mutualism. *Tolerance to cheating, calculated as the maximal value of $\bar{\Delta} \times \Omega$ leading to positive effects on network persistence in average, as a function of proportion of innovative cheating (Ψ), cost associated with mutualism (Λ) and the scenario of simulations, cheaters are generalist species or specialist species. Here results are presented for initial values of connectance φ=0.4, and results are averaged over the 500 initial conditions and over all values of $\bar{\Delta}$ and Ω. Error bars are the standard errors associated with average values. The data underlying this Figure can be found in* https://doi.org/10.5281/zenodo.10102438.

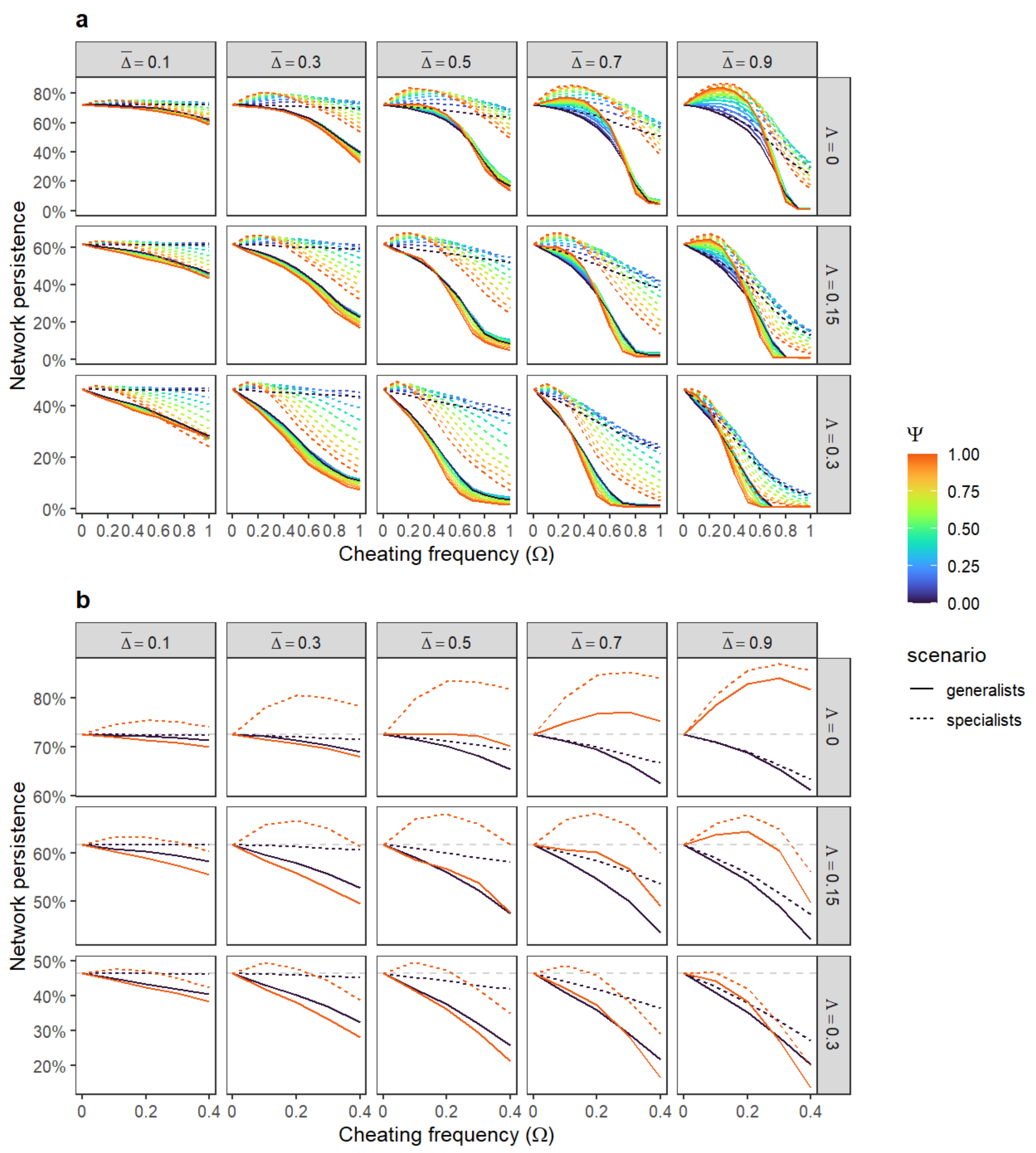

Figure D: Same figure than Fig. A but with a lower value of initial connectance (φ=0.2). *(a) Network persistence as a function of cheating frequency (Ω), proportion of cheaters ($\bar{\Delta}$), cost associated with mutualism (Λ) and proportion of innovative cheating (Ψ). Dashed lines show average results when cheaters are the most specialized species and solid lines when cheaters are the most generalist species. Here results are presented for initial values of connectance φ=0.2 and averaged over the 500 initial conditions. (b) Shows the same than in (a) but on a truncated axis of cheating frequency and for two extremes values only, when cheating is conservative only (Ψ=0) or innovative only (Ψ=1). In (b) the dashed grey line shows the average network persistence when there was no cheating. The data underlying this Figure can be found in https://doi.org/10.5281/zenodo.10102438.*

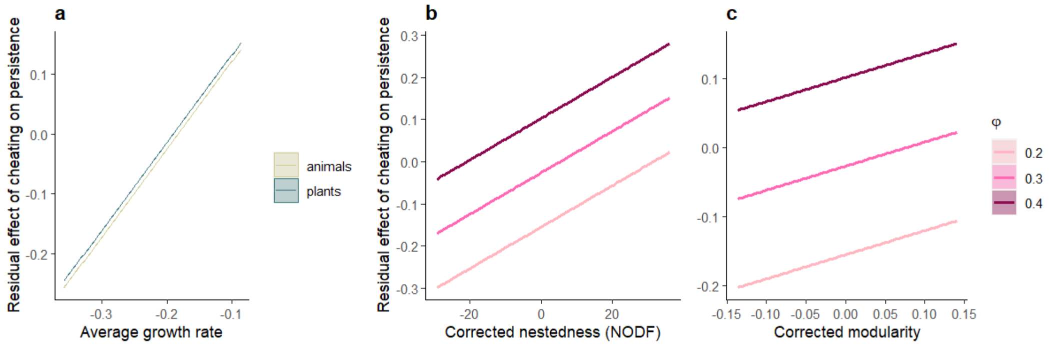

Figure E: Effect of cheating on persistence is linked to plant-pollinator dependency and to network structure. *Residual effects of cheating on persistence as a function of (a) average plants and animals' growth rates, (b) connectance and nestedness, and (c) connectance and modularity of the initial mutualistic network (I_M, cf. Methods). Residual effects of cheating are calculated by subtracting the average effect corresponding to the respective parameter combination from the effect observed for each individual simulation. Positive values indicate effect of cheating on persistence above average effect while negative values indicate the opposite. In (a), more negative growth rates indicate stronger dependency to the other guild. Lines represent model predictions. Nestedness and modularity measures were corrected by connectance (φ) to disentangle their respective effects. The data underlying this Figure can be found in https://doi.org/10.5281/zenodo.10102438.*

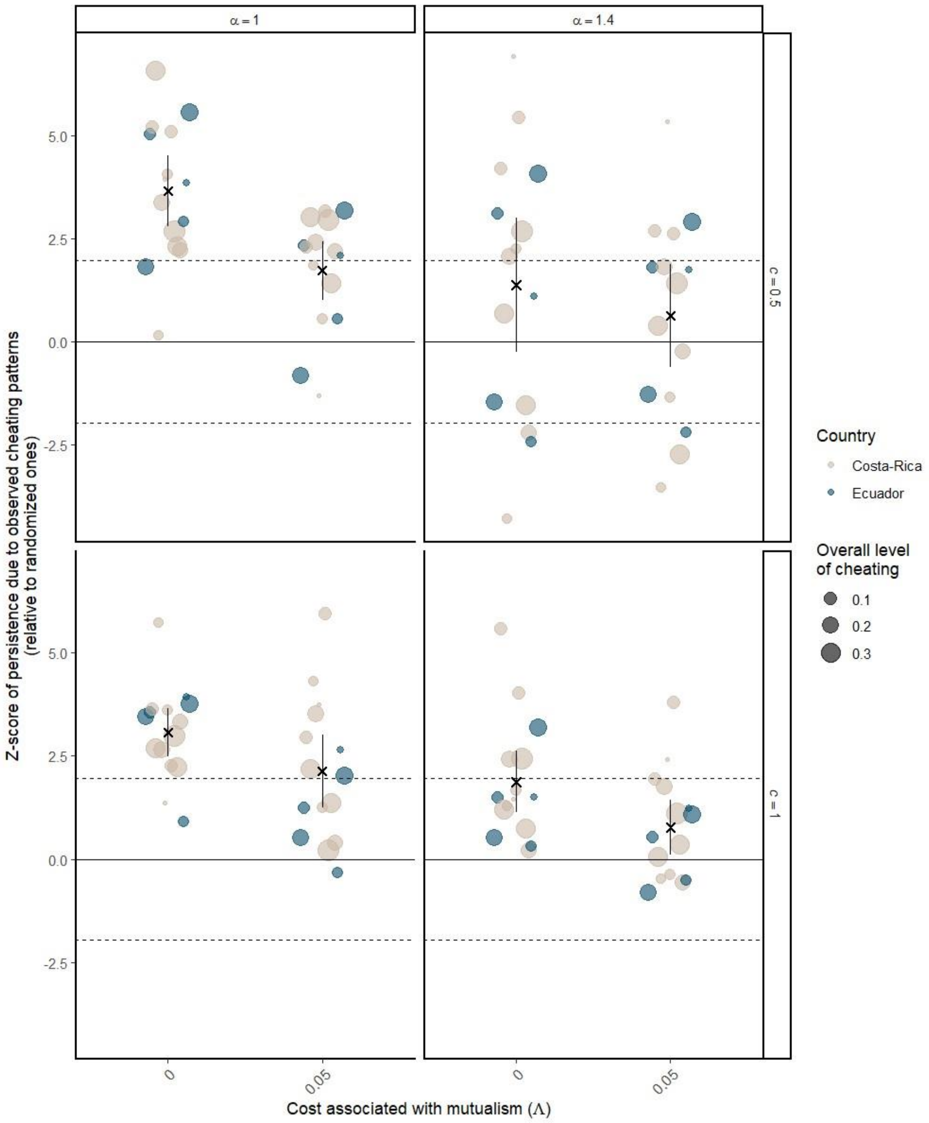

Figure F: Z-score of the persistence when using observed cheating patterns, relative to a distribution of persistence obtained when randomized cheating patterns. *Positive z-score (above 0, solid line) means that observed cheating patterns produce higher persistence than randomized ones, while negative z-score means the opposite. Z-score are significantly positive or negative if they are above or below, respectively, of the dashed lines (1.96 and -1.96). Points represent the average effects over the 100 simulations performed over different growth rate vectors, while error bars represent the standard deviation. Black cross represents the overall average (over sites) and associated 95% confidence interval (vertical error bars, often hidden because too small). The data underlying this Figure can be found in https://doi.org/10.5281/zenodo.10102438.*

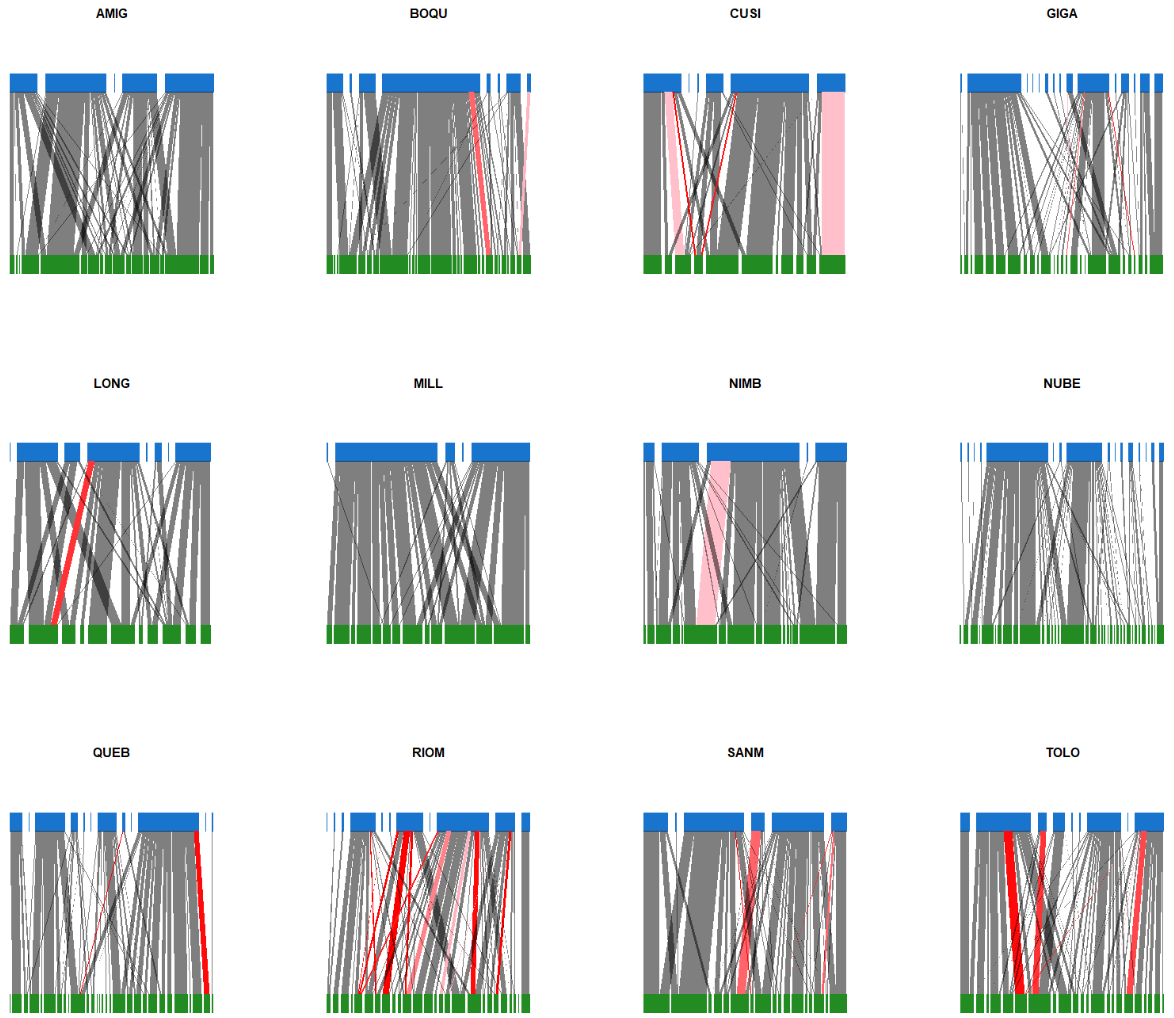

Figure G: Plant-hummingbird interaction network from Costa Rica. *Empirical network of interactions between plants (green) and hummingbirds (blue) from Costa Rica for each site. Thickness of the lines is proportional to interaction frequency, while colour of the lines represents cheating frequency, grey (only legitimate) and from pink (low cheating frequency) to bright red (only illegitimate). The data underlying this Figure can be found in https://doi.org/10.5281/zenodo.10102438.*

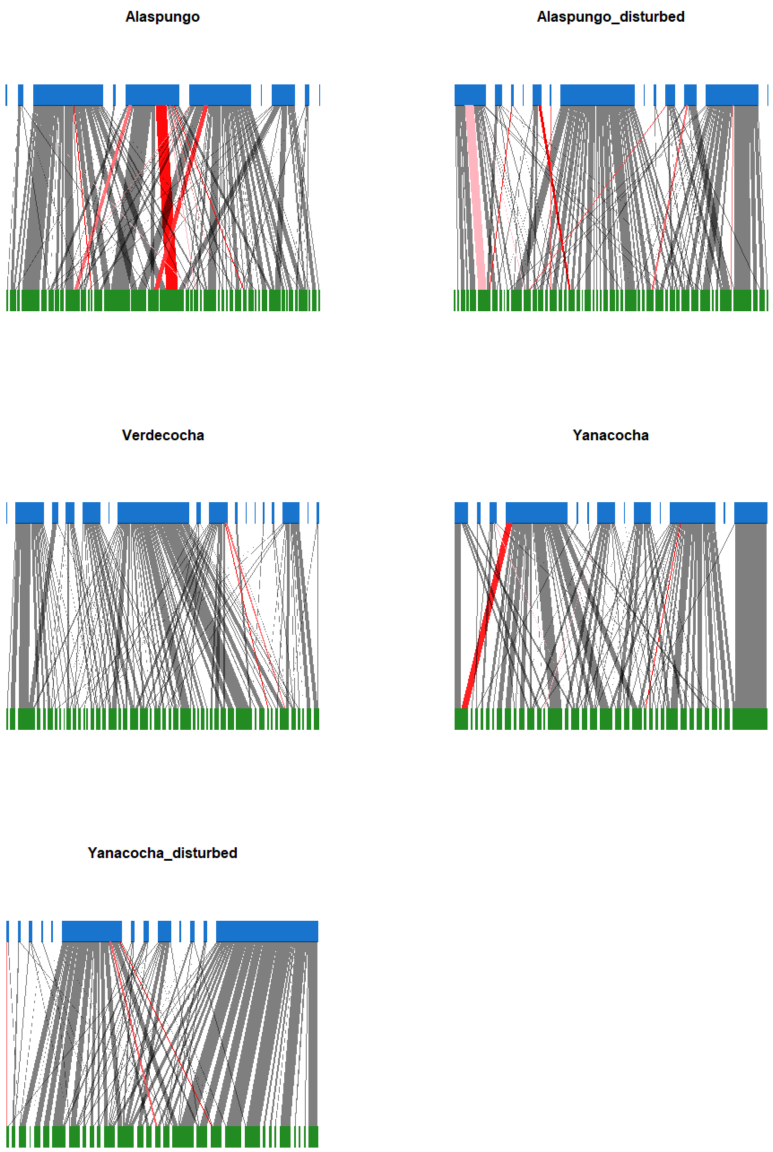

Figure H: Plant-bird interaction network from Ecuador. *Empirical network of interactions between plants (green) and birds (blue) from Ecuador for each site. Thickness of the lines is proportional to interaction frequency, while colour of the lines represents cheating frequency, grey (only legitimate) and from pink (low cheating frequency) to bright red (only illegitimate). The data underlying this Figure can be found in https://doi.org/10.5281/zenodo.10102438.*

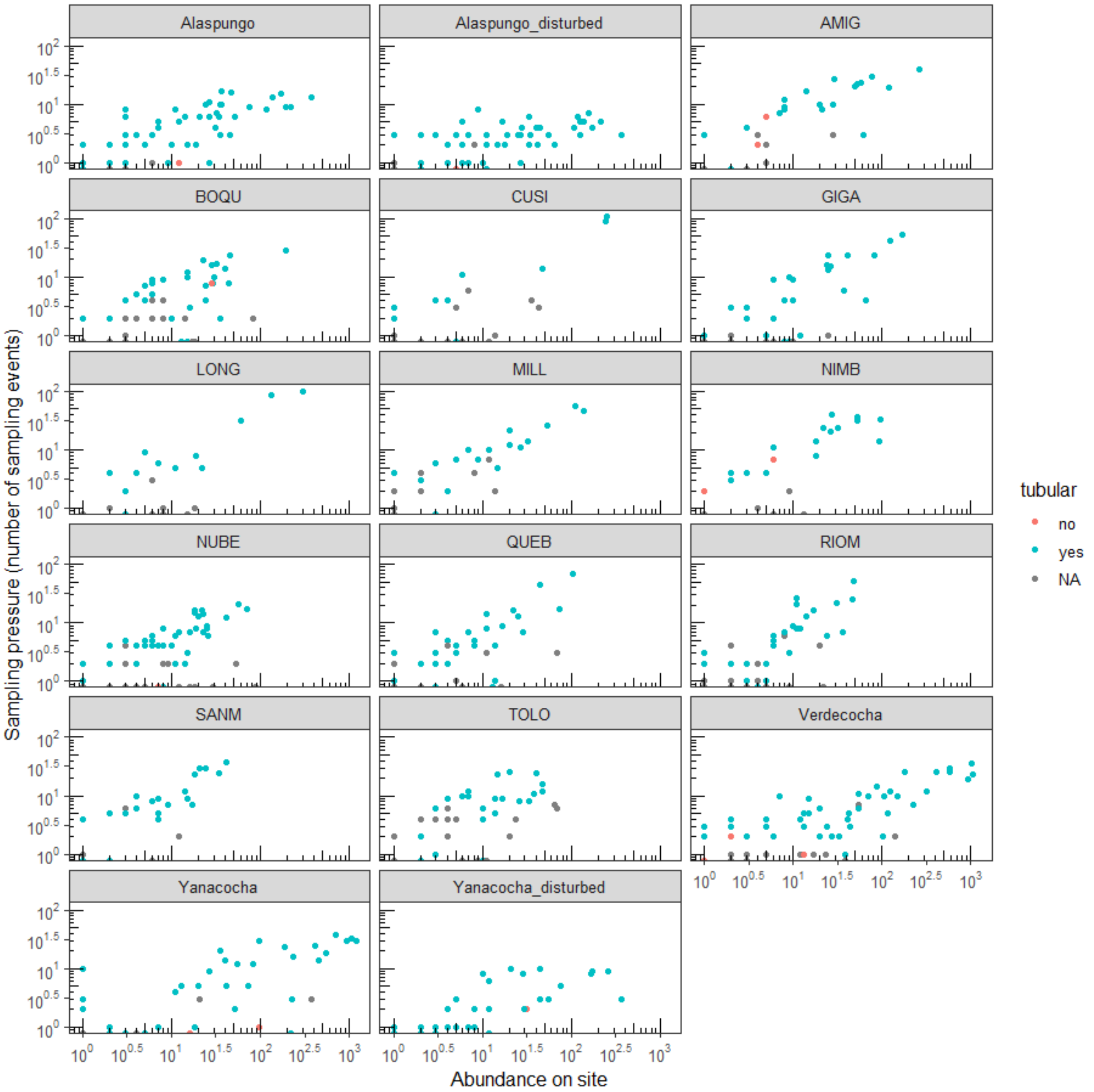

Figure I: Sampling pressure as a function of plant abundance, for each site. *Sampling pressure, the number of sampling events done on one species on a given site, as a function of the abundance of flowering plants on that site. Each point is a plant species. Colors indicate if there is, or not, a closed corolla forming something roughly similar to a tube. NA = non attributed (unknown). Tubular flowers are more likely to be visited by hummingbirds. The data underlying this Figure can be found in* https://doi.org/10.5281/zenodo.10102438.

Supplementary tables

Table A: Results of type II ANOVA of the three models presented in Fig. 4b,d,e. Df = degree of freedom.

Response variable	Explicative variable	χ^2	Df	p-value	Marginal R^2
Frequency of cheating	Diversity (log)	4.2872	1	0.03840	0.11885
	Country	0.0001	1	0.99154	
	Country:Diversity (log)	0.2740	1	0.60066	
Proportion of cheaters	Elevation	4.6810	1	0.03050	0.28708
	Country	3.3014	1	0.06922	
	Country:Elevation	0.1545	1	0.69423	
Overall level of cheating	Elevation	8.9353	1	0.00280	0.46702
	Country	3.1084	1	0.07789	
	Country:Elevation	0.0047	1	0.94531	

Table B: Locations of the studied sites. Ministry of Environment in Ecuador provided the research permit Nº 0162019ICFLOFAUDNB/MAE required to conduct field work.

Site	Longitude_WGS84	Latitude_WGS84	Elevation	Country
AMIG	-83.714	9.547	2778	Costa Rica
BOQU	-83.692	9.484	1993	Costa Rica
CUSI	-83.628	9.330	675	Costa Rica
GIGA	-83.635	9.456	1407	Costa Rica
LONG	-83.488	9.258	644	Costa Rica
MILL	-83.687	9.561	2710	Costa Rica
NIMB	-83.741	9.565	3107	Costa Rica
NUBE	-83.597	9.388	1219	Costa Rica
QUEB	-83.685	9.442	1261	Costa Rica
RIOM	-83.782	9.340	605	Costa Rica
SANM	-83.701	9.500	1973	Costa Rica
TOLO	-83.701	9.474	1681	Costa Rica
Alaspungo	-78.631	0.001	2484	Ecuador
Alaspungo_disturbed	-78.611	-0.010	2787	Ecuador
Verdecocha	-78.598	-0.121	3227	Ecuador
Yanacocha	-78.590	-0.121	3406	Ecuador
Yanacocha_disturbed	-78.586	-0.100	3249	Ecuador
