# Supplementary material for: When cheating turns into a stabilizing mechanism of plant–pollinator communities
Source: PLoS Biol. 2023 Dec 27;21(12):e3002434. doi: 10.1371/journal.pbio.3002434 (PMC10752559; doi:10.1371/journal.pbio.3002434)
Supplement: Attachment — Submitted filename: review_point-by-point-answer.pdf [file pbio.3002434.s003.pdf]

Dear Editor,

We would like to thank you, as well as the reviewers for providing comments and giving us a chance to submit a revised version of our manuscript entitled “When cheating turns into a stabilizing mechanism of mutualistic networks”. Please find attached a point-by-point response to the reviewer comments.

We acknowledge that some motivations of the modelling details were not explicitly argued, especially the choice of modelling diverse communities and the choice of a complex functional response. We account for the reviewers’ comments, mainly by: (1) modifying the introduction to better contextualize our study and provide more methodological inputs in the main text; (2) modifying the methods section to better justify modelling choices; (3) simplifying side analyses to focus the paper on the main message; (4) providing some measure of completeness of the empirical sampling.

We also re-performed all analyses with an updated version of the interaction and trait database, and results were qualitatively the same, changing only marginally the values of estimates. Finally, we performed additional simulations, to investigate the role of diversity and competition in mediating effects of cheating on persistence, as suggested by the reviewers. We integrated these results in a new figure in the main text, to provide a more complete and relevant set of evidence on the positive effect of cheating on network persistence.

To allow reviewers to have access to all modifications we did, we provided as a “Supporting Document” a version of the article with the tracking modification mode. Lines number provided below are the ones for the new version, without the tracking modification mode.

As mentioned in the first cover letter, this article has not been published previously, and it is not under consideration for publication elsewhere, including the Internet. We declare no conflicts of interest related to this work.

Thank you for considering our manuscript for publication. We appreciate your time and look forward to your response.

François Duchenne on behalf of all co-authors

Reviewer 1:

The manuscript by Duchenne et al. combines a theoretical model of a mutualist network and observations of a pollinating bird-flower system to investigate how cheating can evolve at the community scale. Their theoretical models suggest that two routes of cheating evolution (which they term 'conservative' and 'innovative') could be expected to have differential effects on network persistence depending on the makeup of the original network. They then suggest that this would have consequences for which cheating mechanisms are more frequently observed in real systems. They find that hummingbird networks appear to match their predictions.

This paper is highly ambitious and investigates an interesting topic - the potential positive effects of cheating as an example of how the impact of processes can change across scales. It contains lots of interesting novelty, is well written and appears to be well carried out, although I could not access the raw code and data. That said, in my opinion the empirical part of the paper with its clear hypotheses is considerably stronger than the modelling part, where I somewhat struggled to discern the objectives (and this is speaking as modeller!). Nonetheless, the two parts are nicely tied together. Even if there might be some scepticism about the detail of the models, the empirical results stand as something interesting and worth of explanation.

The concluding claim of the abstract that cheating is stabilising is quite a leap and lots of different definitions of stabilising being melded here. Some more description of the circumstances and mechanisms in which mutualisms aid persistence would be good. By this is I don't mean the parameters used in the model, but what it was about the model (and the empirical system) that led to these results (e.g. why destabilising effects of run-away mutualism are avoided).

We thank the referee for taking the time to read and provide constructive comments about our work. We re-wrote part of the manuscript, especially the introduction and methods, to better explain our goals and justify modelling choices. We also provided more inputs in the result section about the kind of statistical tests we used, and we modified the concluding claim of the abstract to make it clearer. Other modifications are detailed below.

Specific points

39- I found the labels of cheating types in abstract not particularly clear. The second para of intro did a much better job and it was only then that I really understood what was intended. The first sentence of the discussion is good too.

We agree, in abstract we were much more constrained by number of words, making the job more challenging. We reformulated the definition of innovative cheating to make it clearer (lines 39-40)

55- grammar?

There were a few errors in this sentence, so we've changed it to correct the conjugation errors.

115 - Appreciate the structure makes modelling papers a challenge, but I would have liked to see some more details of the model form before diving into the results. Linked to my general point, it is hard to shake the feeling that these results are dependent on something about the modelling approach and definition of stability. For example, was it just an effect of connectence? 'conservative' cheating leads to loss of connectence, while innovative adds connectence?

Indeed, we agree that not having the methods before the results can be challenging. We assumed that this format would help researchers not in modelling to get the main assumptions and results without being “scared by equations”, but in the other hand modelling people will have to jump to methods

before reading the results to be satisfied. We tried to provide more information on this point at the end of the introduction and in results.

Indeed, by allowing niche expansion, innovative cheating will tend to increase connectance of the interaction matrix (mutualist and cheating links), while conservative cheating will keep the connectance constant. However, the connectance of the mutualistic matrix (mutualistic links only) decreases evenly with the amount of cheating, regardless the kind of cheating. Thus, different effects of cheating are not mediated by the connectance of mutualistic networks, but by the increase in the connectance of cheating interactions, which allow innovative cheaters to “rely on more partners” as said in the introduction and results. So, this increase in connectance of the interaction matrix under innovative cheating is part of the process we want to study. This idea of niche expansion and gaining partners thanks to innovative cheating but not when cheating in conservative way is now better described in the introduction (lines 72-96).

Linked to this, the 11 million parameter combinations tested seems excessive, but is still ultimately one model form and approach.
11 million simulations can seem like a lot, but with 4 parameters of interest to test, and few sensitivity analyses, for example three different values of connectance, the number of simulations increases quickly. But we agree, at the end even if, we think we explore quite well the parameter space, our simulations are still from only one model, so we did not explore the space of “modelling choice”, that could affect the results. But we did our best to explore the possible cases according to this model.

Why was cheating relatively rare? If defined as avoiding costs, why are most of the interactions non-cheating, costly interactions.

The answer to this question is likely in evolutionary dynamics, which we do not model here. Thus, on this point we can only speculate. It is what we do lines 309-326. Partner selection (avoiding cheaters) is for example a mechanism that has been thought to explain why not all species shifts to cheating (Wechsler & Bascompte 2022).

135 - 'on average'?

Thanks for the correction, we changed “in average” to “on average”.

138 - 'buffer'

Thanks for the correction, we changed “buffers” to “buffer”.

186 - using symbols in this key hypothesis sentence is a bit alienating.

This paragraph functions as a brief introduction to the methods since they are located at the end of the main text. Thus, our decision to incorporate symbols of parameters along with their explanations in this paragraph aims to improve the reader's understanding of the figures. At present, we have opted to maintain this sentence as is, but we are open to making adjustments if the editor deems it necessary.

Statistics and interpretation of Fig 4 -

a) It was hard to know what to take from the image here. An interaction matrix representation may be more clear, but if idea is just to be like 'we made some networks' it should be fine (if a little pointless). Sorting the species to reduce overlap would help a lot too.

Indeed, this figure is here to exemplify the kind of networks we have, the asymmetry in our networks (more plant species than hummingbird species) and the proportion of cheating links (red) vs mutualistic links (black). But there is no important message to take home from this panel, we think it is however good to show to the reader what was the kind of data we started from to produce results presented in other panels.

b) Very hard to trace out what the statistical tests behind this actually are. 'First, cheaters tended to be specialist species' suggests a binary response, but the figure is continuous response (frequency of cheating). Was this a chi-squared test of including log-number of partners?

Sorry, we realized it was not obvious that the chi-square values were from a type II ANOVA performed on a GLM, for each panel. We added a sentence in the results to precise it and refer to Methods for more details (lines 213-215). We also provide more details about that test in the answer, a few lines below.

Why the log-transformation? Not a criticism, but good to know if the result is depend on the transformation - 0-35 isn't an enormous spread. Maybe also specify in the graph axis that it is mutualistic partners?

The log-transformation has the goal to separate better specialists from generalists. Basically, all species that have a high number of partners are considered generalists, so we want them to have close values of generalism (log of the degree), while we consider there is an important difference between a species having one partner and a species having three partners. So, we used the log-transformation to implement a saturation to generalism. We could have classified species in two categories (specialists and generalists) using their number of partners, but his would have require using a threshold, so would have involved another arbitrary choice. We added this explanation to the Methods (Lines 511-513).

The grey-shading here suggests a positive relationship lies within the 95% confidence interval for at least one of the countries but it is hard to read. Need to spell out a little more precisely what tests were done here rather than relying on the SI table. Was there a strong correlation in the predictors?

From what we are aware of, the fact that “a positive relationship lies within the 95% confidence interval” does not inform about the statistical significance or non-significance of the trend.

Otherwise, as described in Methods all lines and confidence intervals are from Generalized Linear Models (GLM), one for each panel (b, d, e), according to the response variable we want to study.

In (b), we used a generalized linear mixed-effects model with a binomial error distribution and a logit link function. This model explains cheating frequency of each bird species in each site per the logarithm of their diversity of mutualistic partners, in interaction with the country. So there is only one quantitative predictor (logarithm of diversity of mutualistic partners), so no problem of correlations between predictors. To test significance of each variable, we performed a type II ANOVA, using indeed Wald Chi-square tests. The correlation of a predictor with the response variable is considered as significant if p-value given by the ANOVA is <0.05.

As said above, we added a sentence in results to clarify the statistical tests and referred to the methods for more details (lines 213-215).

Lastly, as much as I can believe the results are statistically significant, it would be good to have some sense of how much of the variation in the data is explained by the key driver in each case. Is this something that is only just detectable, or something that really controls the structure of the mutualistic networks.

That’s why we plotted the data (points) behind predictions lines. Like this, readers can see if predictions explain completely the data (points on the line) or if the percentage of variance explained is weak (points spread widely around the predictions, lot of “noise”). Here, we can see that in (b) the percentage of variance is evidently weak, so that signal is significant in average but is not enough to fully explain the observations. To provide a quantitative information to the reader, we added the r-square of each model to the Table S1.

c) A bit more outline of the method used to determine the mode of cheating from traits would be very helpful. It looks a nice method and could be emphasised some more.

We agree the method we used here would need a bit more emphasis, but if doing so we are afraid to dilute the main message (i.e. the result on cheating). As methods are at the end, we prefer not overloading the reader with methodological details in the main text, so we provided a quick overview of how we define innovative cheating empirically (*i.e.* cheating outside of the niche defined by the morphological traits of their mutualistic partners) and referred to Methods for more details.

215 - I appreciate the objective of 'closing the loop' here, but as it stands I found it quite hard to follow figure 5 here, and I'm not too convinced how tight a match the model would be to the real system.

As said to referee 2, we acknowledge that simulations parametrized by empirical interactions have many limits. However, we really think that using the structure of empirical networks, number of hummingbird and plant species, distribution of legitimate and illegitimate interactions, and showing that a positive effect of cheating on persistence is likely in this context, is a step more to study how cheating affects persistence of empirical communities. We first have shown that cheating can have positive effects on persistence in some parameter windows. Second that empirical systems appeared to be in that parameter window. So, we want to know what happens if we constrained the model using empirical interaction matrix. But indeed, the model neglects many things that happens in nature and thus in those communities, but we think this fact does not remove value of trying to merge models and data through parametrization.

418- These are big impressive numbers, but it would be still be good to give some sense of network completeness here or how robust the results are to bootstrapping the observations. As this dataset has been used repeatedly, I assume this must exist somewhere already so shouldn't require much further work.

Thanks for this comment, to answer that and other referee's comments, we added a description of the completeness of our sampling in the methods and a supplementary figure (Fig. S8):

"*Since on the transect plant abundance have been recorded independently, we were able to compare the diversity of flowering plants we sample relative to the observed diversity on the transect. On average we sampled 74% of the flowering plants present on each site, with values going from 57% to 93%. Most of non-sampled plants were either non-tubular, so unlikely to be visited by hummingbirds, or species of low abundance (Fig. S8).*" (lines 467-471)

S7 & S8 are near enough useless - too low resolution to read lines, no species identities, and species are not sorted to minimise cross-over.

The main goal of these is to provide a visualization of the distribution of cheating (red links) for readers who would like to have a visual overview of it, and not to provide detailed information on species identities and its interaction links, which can be obtained from the data. It might not be obvious, but species are sorted to minimize cross-over, using "cca" method implemented in *bipartite* R package.

Are the locations presented in Table S2 really millimetre accurate? I'm not clear what is going on here, but has some transformation been applied? It might be worth checking.

Thank you for noticing this. The locations are not millimetre accurate. We extracted the middle point of the transect monitored on each site, which give an artefactual precise number. We corrected that by rounding the coordinates.

Reviewer 2:

Summary: Duchenne et al.'s manuscript explores the impact of inter-specific cheating on multi-species communities, with a focus on communities of plants and pollinators . The manuscript consists of two 'chapters':

◦ In the first chapter, the authors develop a modified Lotka-Volterra model and then conduct simulations exploring the effect of different types of cheating (conservative and innovative) on community coexistence across various parameter combinations. The authors argue that these different types of cheating have different qualitative impacts on community coexistence, with innovative cheating maintaining diversity under certain conditions.

◦ The second chapter analyzes an empirical dataset of observed pollination events involving diverse flowering plants and birds from Costa Rica and Ecuador. The authors claim that the observed cheating patterns in this dataset align with the conditions predicted by the model to promote community diversity via innovative cheating.

This question of whether inter-specific cheating can contribute to maintaining diversity in complex communities is of interest to a wide variety of researchers including community ecologist, evolutionary ecologist and behavioral ecologist. To my knowledge the distinction made in this paper between conservative and innovative cheating is novel, and I found the justification for this distinction based on empirically observed plant-pollinator interactions to be compelling. Because the specific analysis of plant-pollinator interactions is outside of my area of expertise, for this review I will primarily focus on the introduction and theoretical portion of the manuscript. This portion of the manuscript includes some interesting ideas and preliminary results, but I have several major and minor comments which I hope will prove useful and that would need to be addressed in a major revision.

We thank the referee for taking time to read and provide constructive comments on our work. We re-wrote the manuscript, especially the introduction and methods, to better explain our goals and justify modelling choices. To account for comments on old Figure 3, we moved it to supplementary material and instead, provided analyses that deeper explores the mechanisms now presented in introduction: the role of diversity and competition in mediating effects of cheating on persistence.

Major Comments

1. The introduction fails to clearly justify the main hypotheses and does not embeds the manuscript within the broader literature on the ecology and evolution of mutualism. Relevant papers exploring cheating's impact on diversity and coexistence are ignored including Yoder and Nuismer 2010, Leinweber, Inglis and Kummerli 2017, and Heath and Stinchcombe 2014. The authors do not need to be systematic, but considering a wider body of work may help them clarify several claims in the introduction which are overly simplistic and/or unsubstantiated, for example: i) invasion of conservative cheaters always leading to the exclusion of mutualistic partners (lines 71-72), ii) innovative cheating leading to competitive exclusion of other cheaters (lines 74-76), and iii) innovative cheating increases coexistence for specialists but decreasing it for generalists (lines 76-82).

Thanks for the references and comments. We read the references proposed by the reviewer and did another round of literature review. We added Leinweber *et al.* (2017) as an example of a study focusing on the consequences of cheating for the ecological stability (Line 59), as this is clearly an interesting paper for our topic.

We have also missed Week & Nuismer (2021), a very interesting reference for our discussion that we now discuss (lines 320-324).

The paper of Yoder and Nuismer (2010) is extremely relevant to discuss evolution of mutualistic networks, but not really cheating, as authors of the paper themselves discuss that:

"*However, mutualism has also been proposed to create diversification via the evolution of nonmutualistic "cheater" phenotypes (Pellmyr and Leebens-Mack 2000; Law et al. 2001; Ferriere et al. 2007). Because our model does not consider such evolutionary changes to the fundamental fitness outcomes of the interaction (i.e., from a benefit of closer phenotype matching to a cost), it cannot directly address the conditions under which the evolution of cheating may promote diversification in these systems.*"

We completely reformulated the paragraph explaining our hypotheses (lines 72-96) to better explained them and include expectations on the role of species diversity in mediating cheating effects on community persistence.

For clarification about the cited points:

We are a bit surprised to see that the referee interpreted our sentence "*When conservative cheating increases, we expect that it decreases network persistence, because a portion of interactions shift from mutualistic, which tend to increase persistence* (Hale *et al.* 2020)*, to antagonistic or commensalistic, while species do not get any additional partners.*" as "invasion of conservative cheaters always leading to the exclusion of mutualistic partners". It is not what we meant, as we did not discuss the evolution of cheating but the direct ecological consequences of cheating. If an individual cheats on a partner, at the same time it cannot interact mutualistically with it, so when cheating frequency increases the number of mutualistic interactions decreases, just because of time limitations.

We were probably not very clear, so, as said above, we reformulated it to be clearer. All the claims cited by the referee are not something we affirmed but hypothetical consequences of cheating for network persistence. However, since the distinction between conservative and innovative cheating has never been explicitly done before, it is hard to substantiate these hypotheses with previous work.

2. The results section starts with simulations involving a complex ecosystem of 40 species, which makes it challenging to determine the precise mechanism through which innovative cheating is promoting coexistence and how it might depends on plant and/or pollinator diversity. Starting with a minimal example (e.g., 2 pollinators and 2 plants) would greatly strengthen the manuscript, providing clearer illustration of the mechanism at play and potentially being amenable to analytical solutions.

Thanks, for this comment that highlight the fact we did not explicitly say why we decided to study diverse communities, rather than simple ones with few species. We did so because we expected that the effects of cheating on persistence would be mediated by indirect effects, such as competition and facilitation loops, that propagate through long paths in mutualistic networks (Guimarães *et al.* 2017; Cosmo *et al.* 2023). These long paths are present only in diverse communities, as for example the longest path of indirect effects among species you can find in 2x2 community, without passing twice through a species, would be of length 3. Thus, we expect that the positive effects of cheating will appear only if diversity is high enough but would be invisible in small communities.

We completely reformulated the paragraph explaining our hypotheses (lines 72-96) to include these expectations on the role of diversity in mediating cheating effects on persistence.

3. The motivation for Figure 3 (lines 189-203) is unclear and appears disconnected from the main question of the manuscript. To enhance clarity, I would urge the authors to either drop this section or work to better incorporate this result into the manuscript by comparing the effects of different network metrics with the network metrics observed in the empirical data.

Indeed, this section was a bit disconnected from the main question, so we moved it to supplementary and replaced it by a section detailing the role of diversity and competition among pollinators in mediating the effect of cheating on persistence. This new section fits expectation presented in introduction and thus fits better in the flow of the main text.

4. The results shown in Figure 5 (lines 215-221) are poorly motivated and not especially convincing. If I understand correctly, the authors are trying to show that when they constrain the model to reflect parameters that can be estimated, the 'un-estimatable' parameters' will alter coexistence in a characteristic manner and tend to have a positive impact on coexistence in the region we expect 'in nature' (line 221).This argument is both convoluted and not particularly convincing given that i) the authors acknowledge the parameters 'have no empirical meaning' (lines 483-484) ii) there are several big assumption made in the parameter estimation (for example the authors are not paramaterising the model using the actual interaction estimates (Figure S7-S8), but only the aggregated statistics, Lines 470-479) and iii) the authors don't actually explain why low per-interaction benefit and costs are expected 'in nature' (line 221). The authors may want to consider removing this section or placing it in the supplement and instead spending more time in the discussion reviewing the actual empirical evidence for a low cost of mutualism 'in nature' (which was already established as important in figure 2).

Indeed, since effects of cheating depend on how cheating links versus mutualistic links are distributed in the networks (innovative vs conservative cheating, cheating frequency, etc), we wanted to constrain our model using empirical interaction matrices to see we observe a positive effect of cheating or not.

We disagree that this is poorly motivated and convoluted. We first have shown that cheating can have positive effects on persistence in some parameter windows. Second that empirical systems appeared to be in that parameter window. So, we want to know what happens if we constrained the model using empirical interaction matrix, even if a full parametrization of the model is impossible. We do not pretend these simulations parametrized with empirical data provide an absolute argument, because of many limitations, but they are one more step on the way to study effects of cheating on persistence of natural communities.

Regarding the range of cost and per-interaction benefits we expect in nature, we developed a bit more clearly why previous work suggest low per-interaction benefits and costs associated with mutualism in pollination systems (lines 243-247).

5. The methods section (lines 314-374) describing the model and simulations requires substantive revision and clarification as it is very hard to follow. A diagram illustrating the parameter sampling and simulation procedure, along with plots showing the impact of different parameters on the interaction matrix, would be extremely helpful. More-over several modeling choices, seems unnecessarily complicated given the fairly simple question the authors are asking.

Thanks for this comment, which helped us to re-structure and explain better our modelling choice. As detailed below, we changed the methods and the main text to account for this comment.

For example:

i) why go through the steps of building the binary interaction matrix from a sampling of species traits (lines 319- 325)

We agree that we could have directly generated a binary matrix, for example, by sampling from a binomial distribution. However, we wanted to be able to generate binary interaction matrix with a structure, meaning, with generalist and specialist species and being able to modify its connectance. Generating a continuous interaction matrix using gaussian trait and converting it into binary using a threshold appeared to us as quite straightforward and simple way to achieve that while having a matrix

that is relevant. Other process to generate such binary interaction matrix that we had in mind were not necessarily simpler so we sticked to this one.

ii) why sample growth rates from a beta distribution with shape parameter sampled from another distribution (lines 342- 344 and supplementary methods)

We also could have sampled growth rate in a uniform distribution. However by using a beta-distribution we can explore more different configurations, as the shape of the beta distribution will define the average value of the growth rates, more or less negative, but also if they are more or less uniformly distributed (cf. figure below). Thus, by using a beta distribution we explore more different distributions than when sampling in a uniform distribution.

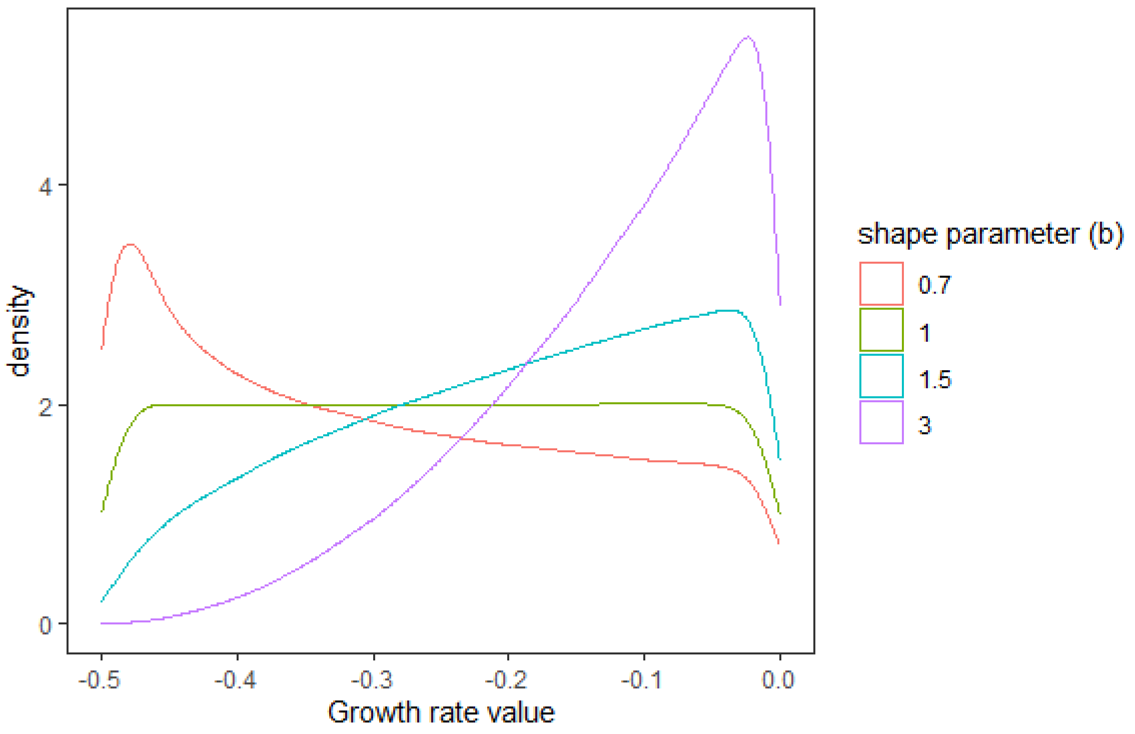

iii) why choose such a complex functional response (supplementary method).

We acknowledge the functional response is a bit more complex than usual generalized Lotka-Volterra models, and it is the core of our model. Since we expect that consequences of cheating on network persistence can be mediated, at least partially, by competition for interacting partners, then we had to use a functional response of type III, including interference. A type III functional response is already quite complex, but we had to add few elements to properly model competition and incorporate cheating.

Furthermore, the vectors of competition (Π_{ik} and Θ_{jk}) or vector of dilution of cheaters among plants sharing cheaters (Υ_{ik}) based on the formula presented in supplementary have a specific purpose. In generalized Lotka-Volterra models, most of the studies model competition strength proportional to the proportion of shared partner, without accounting for the abundance of these partners. In the schematic interaction matrix below (black = interaction, white = no interaction), that means that even if plant species 3 becomes extinct over the simulation, pollinators still compete for it (see figure below). On the contrary, our way to calculate competition vectors account for abundance of partners, so that if a plant species becomes extinct, pollinators will no longer compete for it, and the other way around.

We added a sentence to explain our motivation in Methods (Lines 349-351) and in Supplementary Methods.

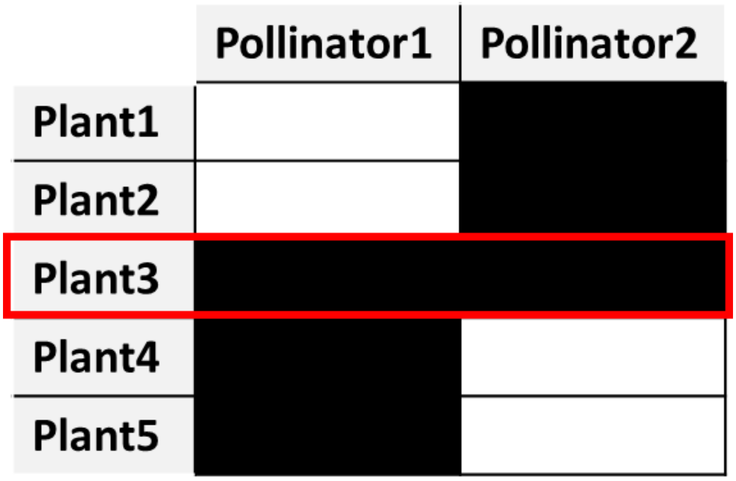

This is the first reason why our functional response is a bit complex.

Second, our functional response also includes the cost associated with mutualism and cheating interaction, in addition to mutualistic ones, that make it obviously more complex than models modelling mutualism only.

Finally, we think this complexity is justified: it makes our model appropriate to model different kinds of cheating in a mutualistic framework and study the consequences on network persistence, accounting for direct costs and benefits for species but also complex indirect effects.

It is unclear if the authors would obtain similar results if they had adopted a simpler approach by say a) sampling a binary interaction matrix directly, b) sampling growth rates from a single distribution and c) using a more simple functional response. If they would not obtain qualitatively similar results then I would be very concerned about the claims they are making given that these arbitrary modeling choice do not naturally emerge from the empirical data.

Since at the end what we put in the model is a binary interaction matrix, the answer to the first concern is surely yes, the step using gaussian trait is just a way to generate a binary matrix that is consistent according to what we know from mutualistic networks (with generalist and specialist species).

For the second concern, since we explore more cases of growth rate distribution using a beta distribution than if we would have used a uniform distribution, then we would say that our results are even more general, as they are based on simulations exploring many possible conditions. There is absolutely no way to estimate these growth rates from empirical data, so we think our approach, exploring many possible values and distributions, better accommodates the uncertainty and is appropriate to make our results more general.

For the last concert, about the functional response: of course, changes in this functional response, which is the heart of the model and concentrate many of our assumptions on cheating, could change the results. As detailed in the previous response, the functional response was carefully thought to be the most adequate to modelling the processes we are interested in.

Minor Comments

1. General: The authors repeatedly refer to 'illegitimate' and 'legitimate' interactions, a vocabulary that I found to be unnecessarily confusing. Wouldn't 'cooperative interaction' and 'cheating interaction' suffice?

Thanks for this opinion. I think this terminology really depends on people background. For people from modelling and evolution we agree that 'cooperative interaction' and 'cheating interaction' would be probably clearer, but in pollination and mutualistic network studies, the terms 'legitimate' and 'illegitimate' are already well established (e.g. Mesquita-Neto *et al.* 2018). So, we wanted to adopt this existing terminology, which is used in many studies on cheating in pollination ecology.

To be clearer we added a definition of what is a legitimate interaction lines 62-63, as only illegitimate interaction was defined before.

2. General: On a related note the terms 'network persistance' and 'stability' and are used rather loosely throughout the manuscript and could be dropped for the sake of precision and clarity. In all the simulations the authors are specifically talking about coexistence and diversity (i.e % species coexisting at equilibrium), and this should be reflected in the text.

We agree that we sometimes used the term stability while it would have been clearer to use persistence, so we changed the term 'stability' into 'persistence' throughout the manuscript. Persistence is indeed a measure of how many species can coexist in a stable way, but since network/community persistence is a well established terminology in ecological networks (Stouffer & Bascompte 2011; Kéfi *et al.* 2019; Barbour *et al.* 2022), we think is relevant to adopt this terminology to facilitate the link between similar works.

3. Lines 50-57: The introduction and abstract would be strengthened by discussing mutualism in a broader phylogenetic context (i.e not just plants and pollinators)

We agree we could have written a more general introduction, but we think that using a specific case of mutualism, which fits our study system, helps readers to understand better the mechanisms as it is illustrated with concrete examples. However, in the first part of introduction we stay very general and in discussion we also generalize our results by talking about mutualism and only pollination. We added a sentence in the discussion to highlight the generality of our theoretical results (lines 270-272):

"*Since cheating has been reported in many kinds of mutualisms, such as mycorrhizal (5), cleaner fish-client (31), or seed-dispersal (7) interactions, it could be a common mechanism driving ecological stability of natural communities.*"

4. Lines 90-91: - The authors need to be much more explicit here and in the abstract about what aspects of the empirical dataset are new for the paper and what parts have been published in previous work. I have gone over the methods multiple times and it is still unclear to me if the authors are analyzing completely new camera trapping data (which would be extremely impressive), whether they are re-classifying interactions recorded in previously collected videos as legitimate or illegitimate (still impressive) or whether they are simply re-analyzing previously collected data.

The dataset has been collected for other purpose and part of it have already been studied (Duchenne *et al.* 2022; Guevara et al. 2023), but here we re-classify interactions recorded in previously collected videos to perform these analyses.

5. Figure 2D: - The authors should also show the volume of parameters space in which cheating decreases 'persistence', given that the claim is about net effect.

We forgot to say that the full parameter space has a volume of 1, since the 3D parameter space presented here is defined by three axes that are proportion,. We added this precision in the legend of

Figure 2. That means that if the volume of the parameter space in which cheating has a positive effect on network persistence is 0.5, cheating is positive for persistence in 50% of the possible parameter combinations on average, and negative for the other 50% of the possible parameter combinations.

6. Line 153-159: This paragraph is very speculative with respect to actual ecological mechanisms at play. But the authors have the full model and so should be able to describe precisely what is going on in their simulations. For example could they not test whether there is competitive exclusion by cheaters due to niche overlap by looking at the effect on pollinator diversity versus plant diversity (rather than simply looking at overall species diversity at equilibrium). If the mechanism in lines 157-159 is true, naively I would expect a greater drop in pollinator diversity compared to plant diversity.

We agree that it is a bit speculative, but theoretical models of diverse mutualistic communities can be hard to interpret, in this case it is hard to interpret because of two main reasons:

- most of the mathematical tools used to study stability and indirect effects are based on equilibrium assumption, so cannot be used to describe what happens on the way to the equilibrium, but at the equilibrium only.

- diversity of plants and hummingbirds are completely coupled. A drop in hummingbird diversity will lead to a drop in plant diversity, as here mutualism is obligatory in both cases. It is possible in some cases to have a drop in diversity in one case without having one in the other case, but this is likely to be rather the exception than the rule. Thus, comparing persistence of pollinators and plants is likely to be non-informative about the mechanisms.

- the importance of indirect *vs* direct effects can be variable and indirect effects are hard to quantify because of mathematical reasons, because the number of possible paths through indirect effects can propagate tend quickly to infinity when network size increase.

However, here we know from the equation that innovative cheating increases competition among species, the remaining questions would be: how important is this effect in mediating the overall result?

So, to investigate the role of competitive exclusion in the fact that innovative cheating strongly affects persistence in a negative way when cheating frequency is close to one, we performed new simulations without competition among pollinators, that we now added to the main text in the new figure 3.

7. Lines 160-174: As stated in my major comments I think this entire section could be removed (or moved to a supplement) and the message of manuscript would be completely unaltered. If the authors choose to keep this section then it needs to be expanded upon because currently there is insufficient detail to understand what the authors are claiming/doing. For example where does the 51% number come from, why does the correlation of residuals to growth rate imply cheating has a larger impact when interaction are weak and why would one a-priori expect different network topologies to differentially modify the impact of innovative cheating.

As said above, this section has been moved to supplementary. 51% of variance comes from the ratio of the variance within parameter combination and among parameter combination. When we do that, we observe that 51% of the variance is explained by parameter combinations (57% with the new simulations).

Since we assume effects of cheating on persistence are mediated partially by indirect effects, which are strongly affected by network structure (connectance, nestedness and modularity), then we expect an impact of network structure on the effects of cheating on persistence. For growth rates, more negative growth rates indicate stronger dependency for species to the mutualistic interactions to persist. Since cheating decrease the direct benefits from mutualism, we expect that when dependency is strong, cheating would have a more negative effects on persistence than when dependency is weak.

8. Lines 215- 221 See previous comment.

9. Figure 5: Is there any reason why the x axis in figure 5 is non-linear? It should be pretty easy to add simulations for 0.2 and 0.25.

It is pretty easy in terms of coding, but simulations are highly time-consuming (they already take weeks using 500 cores on a High Performance Computing system), so we decided to simulate mainly for low and intermediate values of cost, where changes in results can be fast and abrupt, and just to explore one high value of cost to have a view of what happens in this part of the gradient.

10. Figure 5: The authors do not discuss in the main text why they are performing simulations over two different 'strength of competition' (c). If the authors choose to keep this figure, I would only show the results for one set of simulations in the main figure for the sake of clarity (and move the other set of simulations to the supplement)

We used two values of competition strength, because it is a parameter we cannot estimate from the data, we performed simulations for two different values to see how results are affected by competition. We added a brief mention to competition strength (line 241). We did so for every parameter we were not able to estimate from the data, as said in the methods (lines 526-537):

"*Parameters from the functional response could not be inferred from the data: the per-interaction benefit (α), sometimes called mutualistic strength* (Bastolla *et al.* 2009; Duchenne *et al.* 2022), *the cost associated with mutualism (Λ), the handling time (β) and the strength of competition for mutualistic partners (c). To overcome this limitation, we performed simulations over a gradient of possible values, even if it is hard to give an empirical meaning to those parameters. We thus perform simulations from low ($\alpha = 1$) to high per-interaction benefits ($\alpha = 2$) by steps of 0.2, for 3 different values of cost associated with mutualism ($\Lambda \in [0, 0.05, 0.1, 0.15, 0.3]$) and two different values of competition, intermediate (c = 0.5) and high competition strength (c = 1). Since changing the value of β is similar than changing α, we decided to keep β constant.*"

11. Lines 238-239: 'always' seems a bit strong given that there is a region in figure 2D where conservative cheating has a positive effect.

Indeed, there is very tiny region in which conservative cheating can have a slightly positive effect on network persistence, so we changed the "always" to "almost always".

12. Lines 245-250: The authors could emphasize this point more (especially in the introduction). I found the connections to the rich empirical dataset to be the biggest strength of this paper and wish the authors had done more to leverage the data shown in figure S7 and S8.

We agree the empirical part was not really emphasised in the introduction, so we emphasised more our empirical analyses by mentioning them in the introduction (lines 106-112 and 128-133).

13. Line 295: Citation needed

We added a reference to Loeuille (2010)

14. Line 300-302 the lack of additional cost to the plant of cheating is a major assumption of the model (line 111-112) and needs more justification/discussion. How might this additional cost impact the main theoretical predictions in Figure 2. Is it possible to get some idea of whether innovative cheating interaction have additional cost from the camera trap data (damage to flower etc).

We agree that this is an assumption that could change the result in Figure 2, since it would add a negative effect of cheating on persistence of plants. However, from our observations, after piercing, flowers are rarely damaged in visible way, the hole is even hard to see sometimes. The resolution of our picture is not good enough to estimate the damage on flower but the figure 1A of this article (https://peerj.com/articles/9561.pdf) provides a good overview of how piercing damage flowers.

15. Lines 314-387. The authors should make sure they define every parameter before or when they are referencing it (for example in Line 332 I don't think alpha has been defined). Similarly the authors should also make sure they do not define parameters or objects that are not strictly needed to explain the model and/or simulations (I.e the second matrix in line 324-325).

Indeed, thanks for that, we checked and defined parameters on their first use.

16. Lines 363-374 The authors should clarify what coding language the model was written in, what packages were used (and the version of all relevant software).

All the simulations and statistics were performed under R (v 4.3.0), excepting for theoretical simulations done under v3.6.3 on a cluster. This information is also available in the codes, now available on github.

17. Table S1 : Every parameter value not varied in the simulations needs to be justified or mentioned at some point in the methods or main text.

We added a justification of our choice for the three parameter values that did not vary (α, β *and* $c_{P_{ik}}/c_{A_{jk}}$), lines 399-402 and Supplementary Methods.

18. Lines 475-479. I struggled to understand this point, could the authors not just have converted the quantified interaction networks shown in Figure S7-S8 into a binary matrix based on presence/absence of an observed interaction?

Thanks for pointing out that our point was not clear. It is basically what we did, and then, when there was at least one interaction between two species, we estimated the proportion of cheating (illegitimate) and mutualistic (legitimate) interactions. We reformulated this part to be clearer.

Reviewer 3:

Duchenne et al., do a good job of exploring the impact of cheating in mutualistic networks using a combination of Lotka-Volterra population models and bipartite network representations of plant-pollinator interactions. Overall, the manuscript is well-written and effectively explained in most areas, but I would suggest some revisions and additions before considering it for acceptance.

Major Suggestions

One important improvement would be to provide more information about the authors' modeling framework in the introduction section, before delving into the results. Clarifying how their approach differs from previous works will help readers better understand the significance of their research. Additionally, in the results section, adding more details about the model would enhance the reader's comprehension.

Furthermore, the Methods section could benefit from a bit more elaboration. Currently, it may appear dense to a first-time reader, so enhancing its flow within the text would be advantageous. While the provided table is useful, some information may require going back and forth between sections, so integrating pertinent details (including biological insights) directly into the text would improve the overall accessibility of the content.

We agree the Methods section can be dense for the first-time reader. Following this comment and other referees' opinions, we simplified some parts of the manuscript. Specifically, we removed some side analyses presented in supplementary (measure of total effects and inference of missing traits) to keep focus on the analyses presented in the main text, that are already complex enough.

One notable concern revolves around the assumption of the cost of mutualism and its applicability in nature. While such an assumption is necessary for any modeling framework, it could have secondary

repercussions on the results. The authors should comment on the potential robustness of their findings to this assumption and discuss its implications on the overall conclusions.

Furthermore, providing more information about the data and sampling completeness would be valuable. Readers would appreciate insights into whether the data collection time and style introduced any biases in recording interactions. This information will strengthen the reliability and validity of the study's outcomes.

Thanks for this comment, to answer that and other referee's comments we added a description of the completeness of our sampling in the methods and a supplementary figure (now Fig. S8):

"*Since on the transect plant abundance have been recorded independently, we were able to compare the diversity of flowering plants we sample relative to the observed diversity on the transect. On average we sampled 74% of the flowering plants present on each site, with values going from 57% to 93%. Most of non-sampled plants were either non-tubular, so unlikely to be visited by hummingbirds, or species of low abundance (Fig. S8).*" (lines 467-471)

Overall, addressing these major suggestions will enhance the manuscript's clarity and strengthen its overall merit for potential acceptance.

Minor comments and questions

Line 35: [Minor] I was confused initially (before reading the MS) about what methods you are actually using - maybe replace the 'theoretical model' with a couple of phrases about the combination of network methods and the generalized Lotka-Volterra model.

Indeed, theoretical model can be a bit blur, so we changed it to "generalized Lotka-Volterra model", which should give an idea of the kind of model we used to most of the biologists.

Line 53: [Minor] Maybe cleaner fish-client fish (interactions) or fish cleaning (interactions)?

Indeed, cleaner fish-client interactions is probably more explicit, we used it.

Line 57: (studies) has -> have

Thanks, sentence modified.

Line 57: (consequences) has -> have

Thanks, modified.

Line 71: [Minor] define persistence for the readers in this specific context

We now defined network persistence at the beginning of the paragraph, as the number of species being able to coexist.

Line 86: [Minor] go ahead and describe your theoretical model in a little more details (even from the beginning of the paper, the readers have no idea what you mean by your model - and what kind of model)

We moved the description of the model to the end of the introduction to account for that comment and provide more details about the model.

Line 108: [Major] Is the cost of mutualism the same for both partners? What kind of cost is it? I assume for simplicity of modeling, you assumed it to be a constant across all species. Exact reciprocity of the cost, I feel, is non-biological. From an energetic perspective, the cheaters are still using energy to gain plant resources, right? So, the cheaters should just have a lower energetic cost but not zero and also the pollinators should have a different cost associated than plants.

Yes, cost associated with mutualism is the same for both partners and fixed over species. Because we have no idea of its empirical distribution a uniform distribution is the simplest way to model it. Indeed, cost associated with mutualism are probably still partially present for cheaters,

Line 112: due to cheating? And remove previous due?

Indeed, thanks.

Line 116: [Minor] The reader still doesn't know how your Lotka-Volterra model is coupled with your network; also you use proportion in all your plots and not percentage for persistence

We used proportions for model parameters, but the persistence itself is plotted as percentage (color scale of Fig. 2a-b).

Line 198-200: [Minor] Please provide citations

These references were provided in methods but we added them also here.

Line 324: you mean Ioij instead of Imij for the first expression?

Indeed, there was a typo, but on the advice of other referees we removed this part that is not strictly necessary to explain the model after wise.

Line 346: Are benefits alpha just some proportionality constant related to successful pollination? How do you think about it biologically?

We are not sure about what the referee means, but the per-interaction benefits are indeed related to successful pollination and feeding. It is the average increase in individual fitness (survival*reproduction, or per-capita reproduction) that a given interaction brings to the species. So, biologically for plants it would be the link between the number of visits and number of successful pollination events. For hummingbirds it would be how much a given feeding event increases in average the survival*reproduction. But since these benefits are embedded in a non-linear functional response, they saturate, so for example, the successful pollination can increase indefinitely with the number of visits.

We reformulated this part to be clearer.

Line 348: Is beta just handling time (as in table) or has a different meaning as well? Also, just mention it in the text what the parameters mean succinctly.

Beta is really a handling time, we added precision in the text.

References

Barbour, M.A., Kliebenstein, D.J. & Bascompte, J. (2022). A keystone gene underlies the persistence of an experimental food web. *Science*, 376, 70–73.

Bastolla, U., Fortuna, M.A., Pascual-García, A., Ferrera, A., Luque, B. & Bascompte, J. (2009). The architecture of mutualistic networks minimizes competition and increases biodiversity. *Nature*, 458, 1018–1020.

Cosmo, L.G., Assis, A.P.A., de Aguiar, M.A.M., Pires, M.M., Valido, A., Jordano, P., *et al.* (2023). Indirect effects shape species fitness in coevolved mutualistic networks. *Nature*, 619, 788–792.

Duchenne, F., Wüest, R.O. & Graham, C.H. (2022). Seasonal structure of interactions enhances multidimensional stability of mutualistic networks. *Proceedings of the Royal Society B: Biological Sciences*, 289, 20220064.

Guevara, E.A., Bello, C., Poveda, C., McFadden, I.R., Schleuning, M., Pellissier, L., *et al.* (2023). Hummingbird community structure and nectar resources modulate the response of interspecific competition to forest conversion. *Oecologia*, 201, 761–770.

Guimarães, P.R., Pires, M.M., Jordano, P., Bascompte, J. & Thompson, J.N. (2017). Indirect effects drive coevolution in mutualistic networks. *Nature*, 550, 511–514.

Hale, K.R.S., Valdovinos, F.S. & Martinez, N.D. (2020). Mutualism increases diversity, stability, and function of multiplex networks that integrate pollinators into food webs. *Nat Commun*, 11, 2182.

Kéfi, S., Domínguez-García, V., Donohue, I., Fontaine, C., Thébault, E. & Dakos, V. (2019). Advancing our understanding of ecological stability. *Ecology Letters*, 22, 1349–1356.

Leinweber, A., Fredrik Inglis, R. & Kümmerli, R. (2017). Cheating fosters species co-existence in well-mixed bacterial communities. *ISME J*, 11, 1179–1188.

Loeuille, N. (2010). Influence of evolution on the stability of ecological communities. *Ecology Letters*, 13, 1536–1545.

Mesquita-Neto, J.N., Blüthgen, N. & Schlindwein, C. (2018). Flowers with poricidal anthers and their complex interaction networks—Disentangling legitimate pollinators and illegitimate visitors. *Functional Ecology*, 32, 2321–2332.

Stouffer, D.B. & Bascompte, J. (2011). Compartmentalization increases food-web persistence. *Proceedings of the National Academy of Sciences*, 108, 3648–3652.

Wechsler, D. & Bascompte, J. (2022). Cheating in Mutualisms Promotes Diversity and Complexity. *The American Naturalist*, 199, 393–405.

Week, B. & Nuismer, S.L. (2021). Coevolutionary Arms Races and the Conditions for the Maintenance of Mutualism. *The American Naturalist*, 198, 195–205.
