# Supplementary material for: When cheating turns into a stabilizing mechanism of plant–pollinator communities
Source: PLoS Biol. 2023 Dec 27;21(12):e3002434. doi: 10.1371/journal.pbio.3002434 (PMC10752559; doi:10.1371/journal.pbio.3002434)
Supplement: Attachment — Submitted filename: review_point-by-point-answer_2.pdf [file pbio.3002434.s004.pdf]

Dear Editor,

We would like to thank you and the reviewers for providing comments and giving us the opportunity to submit a revised version of our manuscript, now entitled “When cheating turns into a stabilizing mechanism of plant-pollinator communities”. Please find attached a point-by-point response to the reviewer comments.

We have implemented all major suggestions of the reviewers and changed our manuscript accordingly. For example, we reworded the title of our manuscript and performed a null model to complement the results of Figure 5. As most of the comparisons between observed and randomized cheating patterns show higher persistence in observed patterns, this analysis strengthens our results that cheating can increase the persistence of natural communities.

Regarding other small modifications, in our simulations performed for Figure 5, two sites were previously included despite having no cheating (or <0.5%). Since it did not make sense to look for the effect of cheating where there was no cheating, we excluded these sites from that part. Their removal did not affect qualitatively the results. We also corrected a small coding error Figure S8 about how the “tubular trait” was encoded, without any important consequences for the main point of that figure.

To allow reviewers to have access to all modifications we did, we provided as a “Supporting Document” a version of the article with the tracking modification mode. Lines number provided below are the ones for the new version, without the tracking changes.

As mentioned in the first cover letter, this article has not been published previously, and it is not under consideration for publication elsewhere. We declare no conflicts of interest related to this work.

Thank you for considering our manuscript for publication. We appreciate your time and look forward to your response.

François Duchenne on behalf of all co-authors

Reviewer 1

I was reviewer 1 in the first round. My line numbers refer to the submitted manuscript (i.e. not the track changes).
In my view, the authors have done an excellent job at revising the manuscript. I remain of the view that it presents interesting and well supported results. While the paper remains undoubtedly highly complex, the authors do a solid job of presenting their results and in this revision the overall narrative of the paper is considerably more clear. I have a few further comments, but they are almost all aimed at improving the clarity further. As such they come with the caveat that much is likely to be personal preference, but hopefully it helpful to the authors to have another view from someone more distant from the work. I've focussed my comments on the figures, as these will be crucial to distilling the complex story being told here.

We would like to thank the reviewer for taking time again to deep in our manuscript and to provide suggestions to improve our work.

42/43 - Not totally clear what is meant by 'in this case' - I think it means 'for innovative cheating', but it could be speed read to mean 'in this simulation'.

Indeed, it was not very clear. We changed "In this case" by "Under these conditions", which refers directly to the conditions enumerated in the previous sentence (line 42-23).

45 - I would suggest getting a couple of key words about your dataset into the abstract, at least mentioning that it is hummingbirds.

Our dataset is composed by plant-hummingbirds (Costa Rica & Ecuador) and plant-flowerpiercers (Ecuador only) interactions, that's why we used plant-bird interactions and not plant-hummingbird interactions. We added a precision on the bird identity "(hummingbirds and flowerpiercers)" (line 45).

53 - 'described'

Thanks, corrected.

58- 'double 'but' in this sentence is not ideal. (maybe switch second to 'although')

Thanks. We changed the first "but" (to however), because the second but is a codified citation signal, "but see".

63 'cost to them' (maybe?)

We see your point, we added to them just after because, to have to say it only once.

Figure 2 - There is lots of complex information in this figure, and generally I think the authors do a good job at wrestling the multi-dimensional data into some order. One aspect I would question however, is quite which dimension should be in the prime position in the x-axis of the facets. The results text emphasises the proportion of innovative cheating - perhaps this should be on the x?

We see your point. However, since the cheating can be innovative only if a species cheats we did the choice to present the cheating frequency on the x-axis, and the proportion of innovative cheating on y-axis only, because for us there is a natural hierarchy between them. Readers first check if there is cheating (if yes how much?) and then if it is innovative or not.

I also wonder what the value of including cheating frequency =0? In these situations, the average effect on persistence will always be zero (right?) By including it, it creates a somewhat artificial barrier on the left side of each facet. This is not a functional problem, but it adds to the overall cognitive load of the reader.

Indeed, at cheating frequency = 0 the effect of cheating is zero. It is like a control column, to check if we get what we should get here (control for the stability of the numerical resolution of the model for example).

d - is this a proportion/fraction of the volume?

Since the overall possible volume is one, it is the same in that case: proportional volume will be the absolute volume divide by one. We added this explanation to the figure caption.

176 - > specialists -> specialist

Thanks, corrected.

183 - I would consider highlighting this result in the abstract, that the pattern is dependent on a certain level of diversity.

We have a limited place in the abstract that is already long, explaining why we did not focus on that result there. In the abstract, we added "diverse" to the second sentence presenting the results to highlight that diversity has a role to play here:

"*Innovative cheating can enhance community persistence under some conditions: when cheaters have few mutualistic partners, cheat at low or intermediate frequency and the cost associated with mutualism is not too high. Under these conditions, the negative effects of cheating on partner persistence are over-compensated at the community level by the positive feedback loops that arise in* ***diverse*** *mutualistic communities*." (Lines 40-44)

185 - might be good to comment how long these long-loops are

We changed the wording to echoes the one chose by other authors (https://esajournals.onlinelibrary.wiley.com/doi/10.1002/ecy.3080) that might be easier to understand: "*…which likely propagate through long indirect paths, from species to species*" (Lines 190-191). But we did not develop further as understanding precisely what happens here would require a completely new study.

186 - some agreement issues here.

We slightly changed the sentence to correct some mistakes.

Figure 3 - It took me a little while to get that the lower part of b was a zoomed in (I know it says in the caption, but ideally it should be obvious.) I know it must feel unnecessary, but something like writing 'zoom' on the figure could help smooth the journey?

I was not confident in following what 3b is showing - is each dot a particular parameter combination? (changing only the diversity?) What was the grey area? Just the width of the x-axis zoomed in? It might make more sense to highlight the area zoomed? At the moment it looks like it is an area where something special happens.

If I'm honest, this was also a case there the sheer number of points is not particularly helpful - thinning the data may actually add clarity or perhaps switching to a heat map? As far as I can make out, the story of this plot is quite simple - keeping a 1:1 ratio, and adding some annotations ('small communities impacted more ' / 'large communities impacted more') may tell this story in a crisper way.

Sorry if the figure was not very clear. You got it right, in 3b, each dot is the effect of cheating on persistence for one parameter combination, changing only diversity. The grey area is the zoomed part of the x-axis. We changed the figure to make the zoom clearer and bring precision in the figure caption.

3c - same with the other barplot to be honest, it is really hard to see the '0' cases for the generalists. As this is essentially only 4 numbers being presented, could it just be a little table?

It is hard because it is literally zero. We added this information to the caption.

206 -> specialist species (and elsewhere)

Thanks, corrected.

215 - 'chi 2 = 4,29' Should this be a decimal point '.', or is it describing the degrees of freedom in some way?

Indeed, it should be a decimal point, corrected.

Figure 4.

a) I still don't think the web is helpful as presented, but that is ultimately up the authors... Adding a colour legend for the country would be helpful.

Thanks for your suggestions. We added a colour legend for the country.

We believe that is valuable to provide a visual information of how our raw data looks like, since it is the base for the following panels. Especially when Methods are at the end. Thus, we decided to keep that panel for now, but we are willing to remove it if other reviewers and the editor think it should be removed.

b) In the main text, it is described that the there was no statistically significant effect of country, but the plots show a full model including country effects. While not 'wrong' at all, it does make it harder to work out the conclusions we are meant to draw - essentially to shift to thinking that the lines drawn are just to 'guide the eye' and that the confidence intervals are drawn from a maximal model (explaining the uncertainty of direction in some cases).

Indeed, there was no statistical difference between two countries, that is a strong argument to show that the mechanism we highlight are general, not specific to a location. We chose to keep presenting the two country separately to highlight the fact that both datasets are a bit different in a way that Ecuadorian sites include flowerpiercers, a genus of bird visiting flowers but known for cheating, in contrast the data from Costa Rica.

We agree that then the lines are mainly here to guide the eyes, and that the precise statistical result is described in table S2.

I still, it seems like some of the other reviewers, find the part parameterising the theoretical model with the empirical data to be a relatively weak part of the paper. Again, I don't find t wrong as such, just that there isn't a clear conclusion, and it has the effect of adding yet more complication to a challenging paper. But this is ultimately decision for the authors, and I do understand their perspective on this. Their conclusion on L298 (where in parameter space the real system falls) could perhaps be reached more directly than the current analysis.

310 - is there something to cite in terms of prior expectations?

Not really directly linked to cheating, but often evolution is known to optimize individual fitness through time, not population or community properties, such as persistence (Rohr & Loeuille 2023). But it is more a general background, that’s why we did not cite it in that sentence.

328 - subtleties ?

Thanks, corrected.

340 - can you cite some example previous studies?

We added some citation here (already cited in the introduction), now line 357.

Reviewer 2

The manuscript has improved since the previous submission and the authors have largely addressed the substance of my 1st, 2nd and 3rd major comments. I did find the logic in one of the new sections of the introduction to be hard to follow even after multiple readings but I think this could be addressed with simple changes to the writing and structure, so I have made specific suggestion below. I am happy to leave it to the editor to decide whether the authors are sufficiently clear in any final version.

The authors responded to my 4th comment and to the other reviewers comments re figure 5, by reiterating their justification for this section and it's overall role in the paper. I appreciate the authors clarification but I am still not convinced that they have addressed the substance of our comments; namely that it's not clear what is learnt by this parametrization when the the parameters being varied do not map to measurable features of the system. I understand that the authors want to keep this section of the paper so I have two more suggestion that I think would help clarify things.

We thank the reviewer for taking the time to review our manuscript again and to provide suggestions to improve it. We implemented the reviewer suggestion of a randomized null model and added it to our manuscript (see below for details).

Firstly a more minor suggestion; in line 240-241 the authors should state clearly what information from the empirical network is used in the parametrization. They don't need to go into the full detail they have in the methods (which is now much clearer in lines 518-528), but they should state what features of the model are constrained by the empirical data (i.e. network structure and cheating frequency). Right now I think the scope of this analysis is unclear in the main text.

We changed that sentence to precise what was parametrized by empirical data: species diversity per guild, distribution of interactions and cheating frequency for each plant-hummingbird pair (cf. Line 247-249).

Secondly, in my view, looking at the effect on coexistence whilst varying non-estimable parameters is not sufficient evidence for the claim they are making (Lines 240-241), because there is no reference point against which the parameters values are chosen (even order of magnitude), and there isn't a null model against which the effect on coexistence is being compared (lines 529-533). The authors vary the per-interaction benefit between 1 and 2 and the competition strength at 0.5 and 1 but they could very well have picked any arbitrary interval or scale and this just happened to be a range in which a positive effect is observed. The precise claim that the authors seem to want to make is that the specific network structure and distribution of cheating frequency observed in the empirical network helps promote coexistence compared to some different distribution and a different network structure. To make this claim I think one would have to repeat the analysis on a "randomized" empirical network where cheating frequency and network structure are permuted whilst other parameters are kept constant. If the empirically parameterized model showed higher levels of persistence than this "null model", then I think they can justify their claim that 'that observed cheating patterns increased network persistence, when the cost associated with mutualism was low". Otherwise I don't think this analysis really. proves anything. If both the editor and the other reviewers disagree with my opinion on this, I am happy to be over-ruled as I would prefer not to hold up the entire paper over a single part of the analysis.

Thanks for this discussion and this idea of implementing a randomization of cheating frequencies. We implemented this null model, performed new simulations and added that to the manuscript as Figure S8.

Before detailing this new set of simulations with randomized null models, we would like to highlight that Figure 5 was already including a null model, even if it was not randomization. Indeed, Figure 5 showed effect of cheating on persistence, calculated by comparing persistence when using all observed interactions to parametrize the model or only mutualistic ones (excluding cheating). So, the null model used was the interaction network when neglecting cheating. We clarified this point in the figure caption.

We agree that randomizing cheating frequencies is a good idea that would strengthen our manuscript and provide some additional knowledge. We added that results as a Figure S8 and described the workflow in the last paragraph of the Methods (lines 562-576). The results show that for low cost associated with mutualism and low per-interaction benefits, the parameters that we expect in nature (cf. main text), Z-scores are most often positive and even significantly positives, showing that observed cheating patterns produce higher persistence than randomized ones. The positive effect of observed cheating pattern on persistence relative to randomized ones tends to disappear when increasing the cost associated with mutualism and per-interaction benefits (Fig. S8).

Other Comment:

Lines 72-97: This section of the introduction is extremely important and I appreciate the additional details but I found it to be very hard to follow: A few comments might help.

i) Because the entire section is written in the passive voice it introduces a lot of ambiguity and imprecision. For example: 'The consequences of cheating for the number of species being able to coexist (i.e. network or community persistence) are expected to be mediated by two main factors." - expected by who? It's not clear throughout this paragraph what points are established knowledge and what points are the authors reasoning and hypotheses. Simply using the active voice, i.e. "We reasoned that the consequences of cheating for the number of species being able to coexist (i.e. network or community persistence) should be mediated by two main factors" would fix this and improve the flow I would urge the authors to go through the manuscript and try to eliminate this type of ambiguity throughout.

Thanks for raising this point. We corrected those important paragraphs written at the passive voice.

ii) I appreciate that the additional detail was a response to our previous comments but this paragraph is now somewhat meandering and it may be hard for a reader to follow the logic which I think is critically important for understanding this paper. I think it would really help if it were split into 2-3 paragraphs with more explicit signposting of each part of the hypothesis. It is up to the authors how they want to do it but one possible paragraph structure could be: i) Cheating can impact coexistence by two main mechanisms. ii) The effect of cheating on coexistence may depend on whether it is conservative or innovative iii) The effects of innovative cheating may be mediated by network structure and whether the cheater is a generalist or specialist etc.

We followed the reviewer suggestion, we split this paragraph in three different ones and reformulated some sentences to make it easier to understand (lines 72-100).

iii) Punctuation (i.e. commas) throughout this section are inconsistent which made it a little hard read.

We review the punctuation and slightly modified it to make it clearer.

Reviewer 3

The authors have satisfactorily responded to my comments.

We thank the reviewer for taking time to read our work again.

References

Rohr, R.P. & Loeuille, N. (2023). Effects of evolution on niche displacement and emergent population properties, a discussion on optimality. *Oikos*, 2023, e09472.
